# A New Algorithm for High-Integrity Detection and Compensation of Dual-Frequency Cycle Slip under Severe Ionospheric Storm Conditions

**DOI:** 10.3390/s18113654

**Published:** 2018-10-28

**Authors:** Donguk Kim, Junesol Song, Sunkyoung Yu, Changdon Kee, Moonbeom Heo

**Affiliations:** 1 School of Mechanical and Aerospace Engineering and the Institute of Advanced Aerospace Technology, Seoul National University, Seoul 08826, Korea; donguk319@snu.ac.kr (D.K.); albireo37@snu.ac.kr (S.Y.); 2 Ecole Nationale de l’Aviation Civile (ENAC), 31400 Toulouse, France; junesol.song@recherche.enac.fr; 3 Korea Aerospace Research Institute (KARI), Daejeon 34133, Korea; hmb@kari.re.kr

**Keywords:** cycle-slip detection, cycle-slip compensation, insensitive cycle-slip pairs, high-integrity detection, real-time kinematic (RTK)

## Abstract

Many strategies for treating dual-frequency cycle slip, which can seriously affect the performance of a carrier-phase-based positioning system, have been studied over the years. However, the legacy method using the Melbourne-Wübbena (MW) combination and ionosphere combination is vulnerable to pseudorange multipath effects and high ionospheric storms. In this paper, we propose a robust algorithm to detect and repair dual-frequency cycle slip for the network-based real-time kinematic (RTK) system which generates high-precision corrections for users. Two independent and complementary carrier-phase combinations, called the ionospheric negative and positive combinations in this paper, are employed for avoiding insensitive pairs. In addition, they are treated as second-order time differences to reduce the impact of ionospheric delay even under severe ionospheric storm. We verified that the actual error distributions of these monitoring values can be sufficiently bounded by the normal Gaussian distribution. Consequently, we demonstrated that the proposed method ensures high-integrity performance with a maximum probability of missed detection of 7.5 × 10^−9^ under a desired false-alarm probability of 10^−5^. Furthermore, we introduce a LAMBDA-based cycle slip compensation method, which has a failure rate of 1.4 × 10^−8^. Through an algorithm verification test using data collected under a severe ionospheric storm, we confirmed that artificially inserted cycle slips are successfully detected and compensated for. Thus, the proposed method is confirmed to be effective for handling dual-frequency cycle slips of the network RTK system.

## 1. Introduction

Recently, the demand for high-precision navigation systems employing carrier-phase observations has been growing rapidly for applications such as automated vehicle driving and monitoring, collision avoidance, and intelligent transportation systems [1]. One of the typical techniques to obtain cm-level position accuracy is the real-time kinematic (RTK) technique, which is widely used for geodesy and surveying, however, the RTK has been constrained to short-baselines under 10 km. Over the past few decades, network-based RTK techniques have been studied to enlarge RTK coverage per station from medium to long-baseline, for use by dynamic users. The compact network RTK method developed by GNSS Laboratory at Seoul National University is considered as a candidate solution for land vehicle navigation because it provides cm-level positioning service with fast ambiguity resolution under an extremely low-rate data link [1,2,3,4].

One of the most important issues in high-precision navigation systems including the network RTK is generating the integrity information for ensuring the safety of life. The safety-critical navigation system for aviation applications such as satellite-based augmentation system (SBAS) provides integrity information of its corrections [5], however, the carrier-phase-based system, which provides a high-precision correction for users, has a challenging problem that integer ambiguity must be correctly resolved and validated [6]. Furthermore, cycle slip, which is an instantaneous jump of an integer number of a cycle, is a major integrity threat for carrier-phase observations, even after the ambiguity is correctly fixed and validated. A cycle slip occurs unexpectedly when the receiver’s phase-locked loop (PLL) has a loss of lock during a temporary signal blockage or an ionospheric scintillation [7,8,9]. The cycle slip must be handled at the data pre-processing stage since it induces an error with an unpredictable range, which can seriously affect the quality of high-precision corrections and user position solution [7].

A number of processing methods for cycle slip have been studied over the years. In particular, the cycle-slip detection and repair method using dual-frequency observations was proposed by Blewitt [10], Gao and Li [11], Bisnath et al. [7], Liu [12], and Cai et al. [13]. These popular methods employ two complementary geometry-free linear combinations for dynamic users, the Melbourne-Wübbena (MW) combination and ionosphere combination, since the combined cycle slip can be close to zero and cannot be detected using only one combination. For example, the same size of cycle slips on L1 and L2 frequencies such as (10, 10) cannot be noticed on the MW combination, but it has a detectable value on the ionosphere combination. Similarly, the special cycle-slip pair (77, 60), which does not influence the ionosphere combination, is detected by the MW combination. We call these special cycle-slip pairs insensitive pairs [4,13,14].

Most dual-frequency insensitive cycle-slip pairs can be detected by the MW combination and ionosphere combination. In addition, this detection algorithm can be easily used for static and even dynamic users because of the benefit of geometry-free combination; however, it has critical limitations when detecting small cycle slips. First, these techniques are affected by noise and pseudorange multipath effects even if a smoothing or averaging technique such as a Hatch filter and low-pass filter is applied. In other words, these techniques cannot be processed instantaneously and might fail to detect small cycle slips under high-multipath conditions [13,14]. Furthermore, the ionosphere combination is vulnerable to severe ionospheric storm since the remaining bias of the ionospheric variation makes it difficult to detect small cycle slips [13,15,16]. The probability of occurrence of these insensitive pairs is incredibly small [10], but it is still considered an integrity threat to a system requiring high-integrity performance.

To overcome these limitations, triple-frequency signals, which allow many additional combinations such as the extra-wide lane combination and ionospheric reduced combination, are being emphasized for improving performance [17,18,19,20,21]. Especially, many novel combinations of GPS and BeiDou, which are the currently available triple-frequency signals, have been proposed for cycle slip detection without an insensitive pair even under a high-ionospheric storm [15,16,22]. Despite the many advantages of triple-frequency signals, most of the receivers and GNSS satellites only provide dual-frequency signals, currently. That is, the demand for a cycle-slip detection method based on dual-frequency signals remains very strong.

Considering the limitations discussed above, we propose a novel algorithm for cycle-slip detection and compensation using only the dual-frequency carrier-phase observations without pseudorange. In order for the network RTK to be used as safety-critical systems in the near future, it is necessary to ensure a high-integrity information as well as a high-precision corrections. As a first step to enhancing the network-based RTK system, we focus on the high-integrity detection algorithm for continuously operating reference stations (CORS). In this paper, we introduce two independent and complementary ionosphere combinations: one is the geometry-free ionosphere combination usually used in cycle-slip detection, and the other is a new geometry-based ionosphere combination to replace the MW combination. We name these combinations the ionospheric negative and positive combination, respectively. Song and Kee demonstrated that there is no additional geometry-free carrier-phase combination to replace the MW combination for dynamic users [14]; however, geometry-based combinations can be employed in static permanent stations for generating reliable high-precision corrections. Because our ionospheric positive combination constructs the combined cycle slips with positive signs, it can detect a small cycle slip more efficiently compared to the popularly used geometry-based ionosphere-free combination [23,24]. Furthermore, we apply a second-order time-differenced observation (or acceleration) to reduce the influence of ionospheric delay under severe ionospheric storm regardless of baseline lengths [13,15,25].

The remainder of this paper is organized as follows: in Section 2, we first describe the new detection and compensation strategy with two ionospheric acceleration combinations used as monitoring values (MVs). Then, we verify whether the actual error distributions of the MVs could be well assumed as a normal Gaussian distribution during severe ionospheric storms. This analysis of statistical error propagation and bounding is very important because it is impossible to collect an extremely large number of data sets for reliable integrity statistics [5,26,27]. Finally, we evaluate the probability of missed detection and false alarm, which are the most significant indices of a detection algorithm that indicate the integrity performance [28,29,30]. Many former studies overlooked the probability of missed detection and only analyzed whether the size of the cycle slip was greater than the threshold; however, it is necessary to demonstrate the performance of the proposed method quantitatively for conserving integrity. Furthermore, we introduce a reliable cycle-slip compensation method based on the LAMBDA technique [6,31]. In Section 3, we conduct an algorithm test with actual observations to verify the proposed method under a severe ionospheric storm. We inserted the simulated cycle slips artificially into the raw data and checked whether all of them are successfully detected and compensated for. In Section 4, we present our conclusions.

## 2. Cycle-Slip Detection and Compensation Algorithm

### 2.1. Dual-Frequency Cycle-Slip Detection Method

#### 2.1.1. TDSD Carrier-Phase Observations

In order to monitor and detect phase anomalies such as cycle slips and impulse outliers, L1 and L2 carrier-phase observations should be handled as time differences (TDs) and single differences (SDs) between static receivers. By single differencing the observations between receivers, satellite clock biases are eliminated. The TDSD L1 and L2 carrier-phase observations of reference stations are defined as follows:

(1) $$\begin{array}{l} \delta_{T}\Delta \phi_{1}=\lambda_{1}\cdot CS_{1}-\delta_{T}\Delta I+\delta_{T}\Delta L+\delta_{T}\Delta B+\varepsilon_{\delta_{T}\Delta \phi_{1}}\\ \delta_{T}\Delta \phi_{2}=\lambda_{2}\cdot CS_{2}-\gamma \cdot \delta_{T}\Delta I+\delta_{T}\Delta L+\delta_{T}\Delta B+\varepsilon_{\delta_{T}\Delta \phi_{2}} \end{array},$$

where the symbols $\delta_{T}$ and Δ indicate the TD and SD operators, respectively; ϕ is the measured carrier phase; the subscripts “1” and “2” represent the L1 and L2 frequencies, respectively; λ is the wavelength; CS is an integer cycle slip that may exist in phase; *I* is the ionospheric slant delay on the L1 frequency; and γ is the square of the ratio of the L1 and L2 frequencies. In this process, we can eliminate the geometric distance using satellite ephemeris since we know the precise position of reference stations. Furthermore, the tropospheric slant delay can be reduced using general model such as the Saastamoinen and Hopfield model [32]. The symbol *L* denotes the sum of the geometric distance error and the modeling error of tropospheric slant delay. *B* is the receiver clock offset, and ε is the receiver noise of TDSD observation. The noise level of the TDSD observation $\sigma_{\delta_{T}\Delta \phi }$ is twice that of the un-differenced carrier phase, which is typically measured as approximately $\sigma_{\phi_{1}}\approx \sigma_{\phi_{2}}\approx 2\;\mathrm{mm}$ at receivers of CORS [2,33,34].

The bias components of GNSS error sources, except the receiver clock offset, are known to vary slowly over time. The temporal variabilities of the satellite orbit error and tropospheric slant delay are less than 1 and 0.2 mm/s, respectively. Theoretically, the ionospheric slant delay variation can reached 2 cm/s under ionospheric scintillation-free condition [3,35,36,37]. The recent observed ionospheric velocity was less than 0.5 cm/s during a high-ionospheric storm at a low latitude [38,39]. In other words, the time variation of GNSS error sources is much smaller than the size of cycle slips as long as the sampling time is relatively short (1 s in this paper); however, the change of receiver clock, which is unknown and unbounded, should be eliminated to detect the cycle slips.

#### 2.1.2. Receiver Clock Drift Estimate

The SD drift of receiver clock, which has highly stable oscillator, can be estimated precisely from the average value of each satellite’s TDSD ionosphere-free (IF) carrier-phase combination expressed as follows [40]:

(2) $$\delta_{T}\Delta \hat{B}=\frac{1}{m}\sum_{j=1}^{m}\delta_{T}\Delta \phi_{IF}^{j},$$

(3) $$\begin{array}{l} \delta_{T}\Delta \phi_{IF}=a_{1}\cdot \delta_{T}\Delta \phi_{1}+a_{2}\cdot \delta_{T}\Delta \phi_{2}=CS_{IF}+\delta_{T}\Delta L+\delta_{T}\Delta B+\varepsilon_{\delta_{T}\Delta \phi_{IF}}\\ where\;a_{1}=\frac{\gamma }{\gamma -1},\;a_{2}=\frac{-1}{\gamma -1} \end{array},$$

where the symbols $\delta_{T}\Delta \hat{B}$ indicate the estimated SD receiver clock drift; m is the number of the TDSD IF observation $\delta_{T}\Delta \phi_{IF}$; the superscript j represents satellite index. We must check and screen the float size of cycle slip $CS_{IF}$, which may remain in TDSD IF observation before the clock estimation. In order to remove possible cycle slips without the influence of the receiver clock, triple-differenced observations ∇jiδTΔϕIF are used; ∇ji represents the SD between the satellites i and j. Only the TDSD IF observations satisfying the following condition are used to calculate receiver clock drift. A threshold of 3 times the standard deviation of triple-differenced IF observations is set. The value $\sqrt{8}=\sqrt{2^{3}}$ is due to triple differencing [23]:

(4) $$\left|{}^{i}\nabla^{j}\delta_{T}\Delta \phi_{IF}\right|<3\cdot \sqrt{8}\cdot \sqrt{{\left(a_{1}\sigma_{\phi_{1}}\right)}^{2}+{\left(a_{2}\sigma_{\phi_{2}}\right)}^{2}}\;where\;j\neq i.$$

Assuming the noise levels of the TDSD L1 and L2 carrier phase have the same value (i.e., $\sigma_{\delta_{T}\Delta \phi_{1}}=\sigma_{\delta_{T}\Delta \phi_{2}}=\sigma_{\delta_{T}\Delta \phi }=$ 4 mm), the variance of the estimated SD receiver clock drift can be expressed as follows:

(5) $$\sigma^{2}\left(\delta_{T}\Delta \hat{B}\right)=\frac{1}{m}\cdot \sigma^{2}\left(\delta_{T}\Delta \phi_{IF}\right)=\frac{a_{1}^{2}+a_{2}^{2}}{m}\cdot \sigma_{\delta_{T}\Delta \phi }^{2}.\;$$

#### 2.1.3. Cycle-Slip Detection Using the Ionospheric Acceleration

The TDSD L1 and L2 carrier-phase observations after the receiver clock drift compensation can be expressed as follows:

(6) $$\begin{array}{l} \delta_{T}\Delta {\tilde{\phi }}_{1}=\delta_{T}\Delta \phi_{1}-\delta_{T}\Delta \hat{B}=\lambda_{1}\cdot CS_{1}-\delta_{T}\Delta I+\delta_{T}\Delta L+\varepsilon_{\delta_{T}\Delta {\tilde{\phi }}_{1}}\\ \delta_{T}\Delta {\tilde{\phi }}_{2}=\delta_{T}\Delta \phi_{2}-\delta_{T}\Delta \hat{B}=\lambda_{2}\cdot CS_{2}-\gamma \cdot \delta_{T}\Delta I+\delta_{T}\Delta L+\varepsilon_{\delta_{T}\Delta {\tilde{\phi }}_{2}} \end{array}.$$

We call Equation (6) the TDSD carrier-phase residual. In this section, we describe two L1/L2 linear combinations of carrier phase for obtaining the MVs of cycle-slip detection. The first observation is the geometry-free combination that only contains ionospheric slant delay, which is widely used to detect dual-frequency cycle slips [7,10,11,12,13,25,41]. In this paper, we named it the ionosphere negative (IN) combination since L1 and L2 cycle slips are combined with negative signs:

(7) $$\begin{array}{l} \delta_{T}\Delta I^{-}=b_{1}^{-}\cdot \delta_{T}\Delta {\tilde{\phi }}_{1}+b_{2}^{-}\cdot \delta_{T}\Delta {\tilde{\phi }}_{2}=\delta_{T}\Delta I+\frac{1}{\gamma -1}\left(\lambda_{1}\cdot CS_{1}-\lambda_{2}\cdot CS_{2}\right)+\varepsilon_{\delta_{T}\Delta I^{-}}\\ whereb_{1}^{-}=\frac{1}{\gamma -1},\;b_{2}^{-}=\frac{-1}{\gamma -1} \end{array}.$$

In general, the size of the combined cycle slips in the IN combination is much greater than the ionospheric velocity and the observed noise level; however, the size can be almost zero even if the sizes of L1 and L2 cycle slips are not small. These special cycle-slip pairs are defined as insensitive pairs, which cannot be detected. Therefore, we need a complementary linear combination that can detect insensitive cycle-slip pairs in the IN combination [4,14].

The second observation we have chosen is another ionosphere combination that couples the L1 and L2 cycle slips with positive signs [23]. We named it the ionosphere positive (IP) combination, which can be expressed as follows:

(8) $$\begin{array}{l} \delta_{T}\Delta I^{+}=b_{1}^{+}\cdot \delta_{T}\Delta {\tilde{\phi }}_{1}+b_{2}^{+}\cdot \delta_{T}\Delta {\tilde{\phi }}_{2}=-\delta_{T}\Delta I+\frac{1}{2}\left(1+\frac{1}{\gamma }\right)\delta_{T}\Delta L+\frac{1}{2}\left(\lambda_{1}\cdot CS_{1}+\frac{1}{\gamma }\lambda_{2}\cdot CS_{2}\right)+\varepsilon_{\delta_{T}\Delta I^{+}}\\ where\;b_{1}^{+}=\frac{1}{2},\;b_{2}^{+}=\frac{1}{2\gamma } \end{array}.$$

The IP combination is not geometry-free; however, the variations of satellite orbit error and tropospheric slant delay are negligible in case of stationary observations, as described in Section 2.1.1. Since two L1/L2 linear combinations combine cycle slips with different signs and are complementary to each other, all cycle slips can be detected under quiet ionospheric storm, provided the ionospheric velocity is sufficiently small. As discussed in Section 2.1.1, the change of ionospheric slant delay cannot be ignored for detecting small cycle slips, because it can be greater than 2 cm/s under high ionospheric storm. Thus, we employ ionospheric acceleration, which is the second-order derivative of ionospheric slant delay, as MVs. Since the bias of ionospheric acceleration is less than 10^−4^ m/s^2^ even under high ionospheric disturbance [42], many studies considered it as the MV for robust cycle-slip detection [13,15,25]. The two linear combinations of the ionospheric acceleration can be expressed as follows:

(9) $$\delta_{T}^{2}\Delta I^{-}=\delta_{T}^{2}\Delta I+\frac{1}{\gamma -1}\left(\lambda_{1}\cdot CS_{1}-\lambda_{2}\cdot CS_{2}\right)+\varepsilon_{\delta_{T}^{2}\Delta I^{-}},\;$$

(10) $$\delta_{T}^{2}\Delta I^{+}=-\delta_{T}^{2}\Delta I+\frac{1}{2}\left(1+\frac{1}{\gamma }\right)\delta_{T}^{2}\Delta L+\frac{1}{2}\left(\lambda_{1}\cdot CS_{1}+\frac{1}{\gamma }\lambda_{2}\cdot CS_{2}\right)+\varepsilon_{\delta_{T}^{2}\Delta I^{+}}.\;$$

Equation (9) corresponds to the IN combination, and Equation (10) corresponds to the IP combination. The symbol $\delta_{T}^{2}$ represents the second-order TD operator. These MVs have only receiver noise with negligible biases under the non-cycle-slip hypothesis. Therefore, the cycle slips can be detected when the following condition is satisfied:

(11) $$\begin{array}{ll} & \left|MV^{-}\right|≜\left|\delta_{T}^{2}\Delta I^{-}\right|>T^{-}=K_{FA}^{-}\cdot \sigma_{\max}\left(\delta_{T}^{2}\Delta I^{-}\right)\\ or & \left|MV^{+}\right|≜\left|\delta_{T}^{2}\Delta I^{+}\right|>T^{+}=K_{FA}^{+}\cdot \sigma_{\max}\left(\delta_{T}^{2}\Delta I^{+}\right) \end{array},$$

where $T^{-}$ and $T^{+}$ indicate the thresholds for each MV. The detection thresholds are calculated based on the confidence level $K_{FA}$ and the maximum standard deviation of the MV. We will analyze the noise level of the MV in Section 2.2 and discuss how to determine thresholds for the detection criterion in more detail in Section 2.3.

### 2.2. Error Propagation in Monitoring Values

#### 2.2.1. Theoretical Noise Analysis of Monitoring Values

In this section, we theoretically discuss the noise level in the MVs. We should assume the MVs under the non-cycle-slip hypothesis as a normal Gaussian distribution. Unfortunately, the actual data does not have a perfect Gaussian distribution, because of, e.g., the influence of carrier-phase multipath effects, clock estimation errors, and the remaining bias of ionospheric acceleration. Therefore, the standard deviation of the MVs must be carefully determined for sufficiently bounding the tails of the actual error distribution [5,26,27]. In other words, we should theoretically analyze the statistics of the MVs considering the worst possible scenario.

The general form of the linear combination of the TDSD carrier-phase residual can be expressed by Equation (12), and the variance of that can be calculated from Equation (13). We assume the carrier phase of L1 and L2 have the same noise level:

(12) $$\begin{array}{ll} \delta_{T}\Delta {\tilde{\phi }}_{\left(\beta_{1},\beta_{2}\right)} & =\beta_{1}\cdot \delta_{T}\Delta {\tilde{\phi }}_{1}+\beta_{2}\cdot \delta_{T}\Delta {\tilde{\phi }}_{2}\\ & =\beta_{1}\cdot \left\{\delta_{T}\Delta \phi_{1}-\delta_{T}\Delta \hat{B}\right\}+\beta_{2}\cdot \left\{\delta_{T}\Delta \phi_{2}-\delta_{T}\Delta \hat{B}\right\} \end{array},$$

(13) $$\sigma^{2}\left(\delta_{T}\Delta {\tilde{\phi }}_{\left(\beta_{1},\beta_{2}\right)}\right)=\left(\beta_{1}^{2}+\beta_{2}^{2}\right)\cdot \sigma_{\delta_{T}\Delta \phi }^{2}+{\left(\beta_{1}+\beta_{2}\right)}^{2}\cdot \sigma^{2}\left(\delta_{T}\Delta \hat{B}\right).\;$$

Assuming the biggest value of the variance of the estimated clock drift from Equation (4), the variance of the TDSD carrier-phase residual becomes maximum as follows:

(14) $$\begin{array}{l} \sigma_{\max}^{2}\left(\delta_{T}\Delta {\tilde{\phi }}_{\left(\beta_{1},\beta_{2}\right)}\right)=\left\{\left(\beta_{1}^{2}+\beta_{2}^{2}\right)+{\left(\beta_{1}+\beta_{2}\right)}^{2}\cdot \frac{a_{1}^{2}+a_{2}^{2}}{m}\right\}\cdot \sigma_{\delta_{T}\Delta \phi }^{2}≜func\left(\beta_{1},\beta_{2}\right)\cdot \sigma_{\delta_{T}\Delta \phi }^{2}\\ where\;m=1 \end{array}.$$

Since the carrier-phase measurements can be regarded as uncorrelated in time and each observation, the variance of second-order TD observations is 6 times (i.e., $\sigma_{\delta_{T}^{2}\phi }^{2}=6\cdot \sigma_{\phi }^{2}$), and SD observations is twice (i.e., $\sigma_{\Delta \phi }^{2}=2\cdot \sigma_{\phi }^{2}$) that of the un-difference observations. Therefore, the maximum variance of the MVs of the IN and IP acceleration can be calculated from Equations (15) and (16):

(15) $$\sigma_{\max}^{2}\left(\delta_{T}^{2}\Delta I^{-}\right)=func\left(b_{1}^{-},b_{2}^{-}\right)\cdot \sigma_{\delta_{T}^{2}\Delta \phi }^{2}=12\cdot func\left(b_{1}^{-},b_{2}^{-}\right)\cdot \sigma_{\phi }^{2},\;$$

(16) $$\sigma_{\max}^{2}\left(\delta_{T}^{2}\Delta I^{+}\right)=func\left(b_{1}^{+},b_{2}^{+}\right)\cdot \sigma_{\delta_{T}^{2}\Delta \phi }^{2}=12\cdot func\left(b_{1}^{+},b_{2}^{+}\right)\cdot \sigma_{\phi }^{2}.\;$$

Finally, the maximum standard deviation of the MVs is theoretically determined as follows if we set the carrier phase noise as 2 mm:

(17) $$\sigma_{MV^{-}}≜\sigma_{\max}\left(\delta_{T}^{2}\Delta I^{-}\right)=7.57\cdot \sigma_{\phi }=15.1\;\;\mathrm{mm}/s^{2}\;$$

(18) $$\sigma_{MV^{+}}≜\sigma_{\max}\left(\delta_{T}^{2}\Delta I^{+}\right)=8.52\cdot \sigma_{\phi }=17.1\;\mathrm{mm}/s^{2}\;$$

#### 2.2.2. Actual Error Distribution of Monitoring Values

In this section, we analyze the actual error distribution of MVs by using the observed data under various ionospheric storm conditions. We especially validate that the “fat tails” (i.e., non-Gaussian edge) of the actual error distribution are properly bounded to the theoretical error distribution of the MVs that we determined previous section. That is, we prove the actual error distribution can be replaced the assumption of theoretical normal distribution.

**Figure 1:**
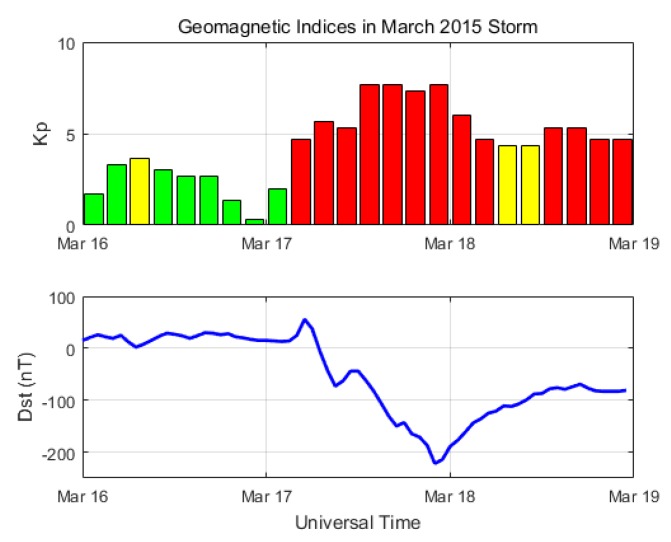
The geomagnetic indices (Kp and Dst) during 16–18 March 2015.

**Figure 2:**
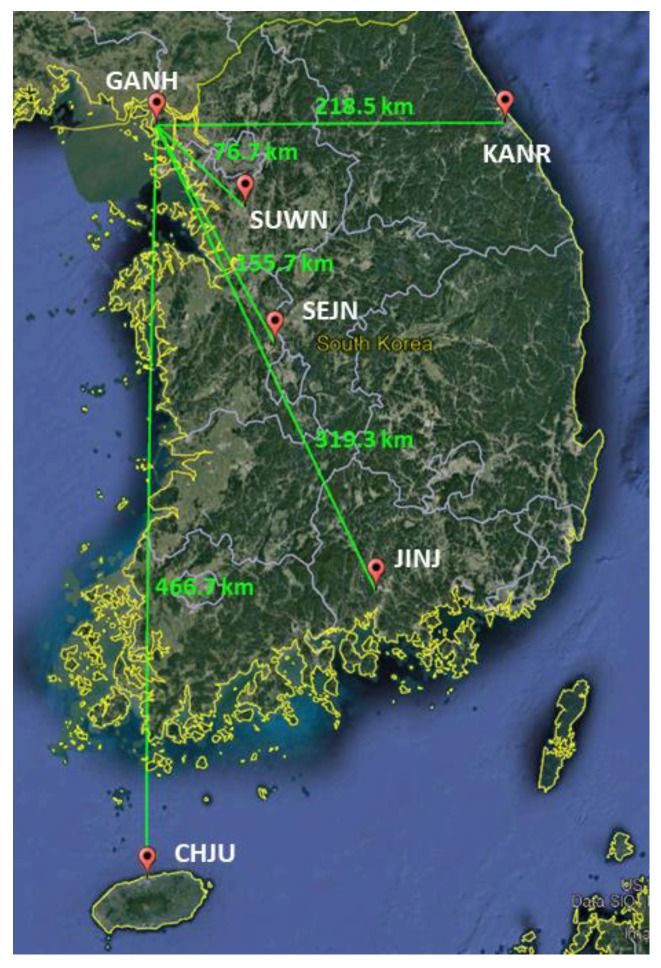
Locations of the six reference stations of NGII in Korea.

A severe geomagnetic storm occurred on 17 March 2015. During this storm, the geomagnetic Kp index reached 8-, and the Dst index dropped to −223 nT [43]. Figure 1 shows the time history of the geomagnetic indices Kp and Dst for 16–18 March 2015. The raw data of geomagnetic indices were obtained from the World Data Center (WDC) for Geomagnetism (Kyoto, Japan). The ionosphere was disturbed violently from quiet to severe for these three days in the mid-latitude including the Korean region. We collected GPS carrier-phase measurements with 1-s intervals for the three days from the six reference stations of the National Geographic Information Institute (NGII) in Korea. The GANH station is chosen as a master station, and five other stations are assigned to auxiliary stations to calculate the SD between receivers. All the stations have a Trimble NetR9 receiver connected with a TRM59800 antenna, and their locations are shown in Figure 2.

We pre-processed cycle slips and outliers in the collected data by using Bernese GNSS software and calculated the IN acceleration and IP acceleration. We call these values the nominal MVs that represent the non-cycle-slip hypothesis. All baseline data of visible satellites that have elevation angles greater than 5° are used for statistical analysis.

Though we collected the almost 10^6^ number of sample data, the assumption of Gaussian distribution cannot be represented by experimental data alone. Therefore, we applied the over-bounding analysis technique that is widely used in aviation area such as SBAS and ground-based augmentation system (GBAS) in order to validate the reliable assumption of the normal error distribution [5,26,27].

**Figure 3:**
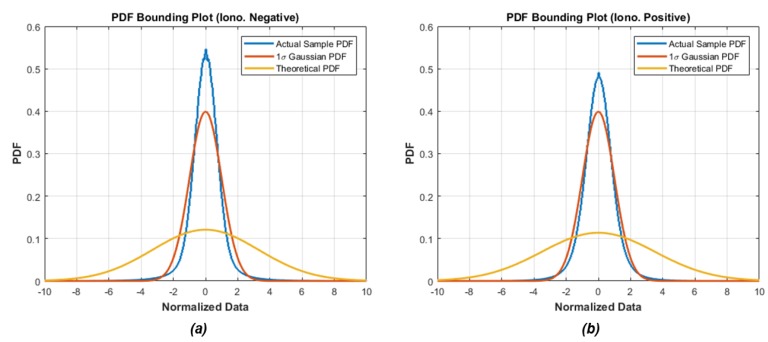
Probability density function of nominal monitoring values: (**a**) ionospheric negative acceleration; (**b**) ionospheric positive acceleration.

Figure 3 shows the probability density function (PDF) of the two nominal MVs. The blue line represents the actual sample-data distribution normalized by the standard deviation of experimental data, and the red line represent the 1-sigma standard Gaussian distribution. As we can see, the red line appears to be a good statistics of the blue line; however, the both tails of actual error distribution exceed the standard Gaussian distribution. In other words, the probability that the actual nominal MV has a large deviation value (e.g., 4-sigma) under the non-cycle-slip hypothesis exceeds the probability that we expected. On the other hand, the yellow line, which represents the Gaussian distribution based on the theoretically determined standard deviation in Section 2.2.1, properly bounded the both tails of the actual error distribution. Because we calculated the theoretical values conservatively (i.e., worst-case), the probability of potential failure condition or called integrity risk make can be made sufficiently small.

The PDF bounding analysis shown Figure 3 can intuitively check the overall distribution of the sample data; however, it is difficult to identify whether the tail of the distribution is actually bounded under the very small probability level such as 10^−7^ since the tail probability is quite sensitive. In addition, it is not possible for bounding all deviation values since its integral over the entire space is equal to one [26]. The cumulative distribution function (CDF) bounding analysis, which overcomes the PDF analysis problem, examines the tails of actual sample distributions to show that they are well-bounded and thinner than the tails of a normal Gaussian distribution [5,26,27].

**Figure 4:**
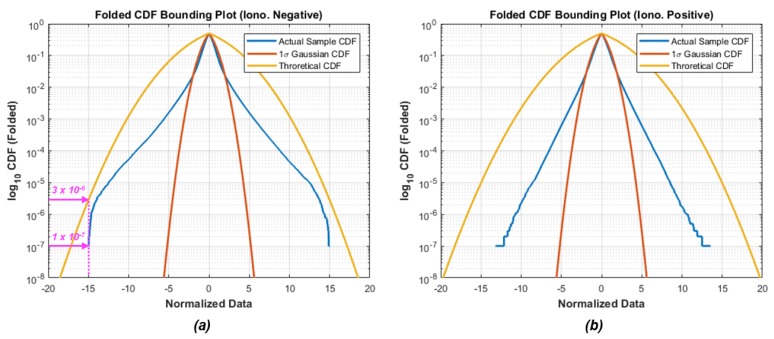
Folded cumulative distribution function of nominal monitoring values (note that the scale of its vertical axis is logarithmic): (**a**) ionospheric negative acceleration; (**b**) ionospheric positive acceleration.

Figure 4 represents the folded CDF bounding plots of the two nominal MVs normalized by the standard deviation of experimental data. The plot of a standard CDF has an S-like shape, while the folded CDF plots, which folds the top half of the standard CDF graph over, has mountain shape [44]. In order to show the tail shape of the distribution more clearly, scale of its vertical axis is logarithmic. The blue curve line represents the actual sample-data CDF, the red line represents the 1-sigma standard CDF, and the yellow line means the conservatively determined CDF in Section 2.2.1. We can clearly see that the blue curve line has non-Gaussian tail, which is fatter than the red line, but thinner than the yellow line. This means that the theoretically determined distribution well bounds the actual sample distributions. For example, as shown in Figure 4a, the probability that the normalized error of actual MVs of IN combination (the blue line) exceeds a 15-sigma value is less than or equal to 10^−7^, under the non-cycle-slip hypothesis; however, the theoretical standard deviation that we conservatively calculated in Section 2.2.1 is designed that the probability of the actual error of MV exceeding a 15-sigma is less than or equal to about 3.0 × 10^−6^. In conclusion, we demonstrate that the theoretically determined standard deviation of the MVs are well-bounded at the 10^−7^-level probability of the normal Gaussian distribution even under severe storms. Table 1 summarizes the statistics of the distribution of the two MVs. The theoretical maximum standard deviation is approximately more three times inflated than the standard deviation of actual data.

### 2.3. Threshold Determination Scheme

#### 2.3.1. Probability of False Alarm and Probability of Missed Detection

In this section, we describe the probability of false alarm and of missed detection, which are the important performance indices of the cycle-slip detection. We already demonstrated that our two independent MVs, the IN acceleration and IP acceleration, could be assumed to have an over-bounded Gaussian distribution with a zero mean and variances of $\sigma_{MV^{-}}^{2}$ and $\sigma_{MV^{+}}^{2}$, respectively in the cycle-slip-free case (i.e., non-cycle-slip hypotheses *H*_0_).

(19) $$H_{0}:MV^{-}\sim N\left(0,\sigma_{MV^{-}}^{2}\right)\;f\left(x|0,\sigma_{MV^{-}}^{2}\right)=\frac{1}{\sqrt{2\pi \sigma_{MV^{-}}^{2}}}\exp\left(-\frac{x^{2}}{2\sigma_{MV^{-}}^{2}}\right),$$

(20) $$H_{0}:MV^{+}\sim N\left(0,\sigma_{MV^{+}}^{2}\right)\;f\left(x|0,\sigma_{MV^{+}}^{2}\right)=\frac{1}{\sqrt{2\pi \sigma_{MV+}^{2}}}\exp\left(-\frac{x^{2}}{2\sigma_{MV^{+}}^{2}}\right).$$

When a cycle slip occurs, the PDFs of the MVs are shifted by an amount equal to the size of the combined cycle slip. We define the cycle-slip hypothesis *H_k_* corresponding to the cycle-slip (or fault) event *k* as follows:

(21) $$H_{k}:MV^{-}\sim N\left(\mu_{k}^{-},\sigma_{MV^{-}}^{2}\right)\;f\left(x|\mu_{k}^{-},\sigma_{MV^{-}}^{2}\right)=\frac{1}{\sqrt{2\pi \sigma_{MV^{-}}^{2}}}\exp\left\{-\frac{{\left(x-\mu_{k}^{-}\right)}^{2}}{2\sigma_{MV^{-}}^{2}}\right\},\;$$

(22) $$H_{k}:MV^{+}\sim N\left(\mu_{k}^{+},\sigma_{MV^{+}}^{2}\right)\;f\left(x|\mu_{k}^{+},\sigma_{MV^{+}}^{2}\right)=\frac{1}{\sqrt{2\pi \sigma_{MV^{+}}^{2}}}\exp\left\{-\frac{{\left(x-\mu_{k}^{+}\right)}^{2}}{2\sigma_{MV^{+}}^{2}}\right\}.\;$$

**Figure 5:**
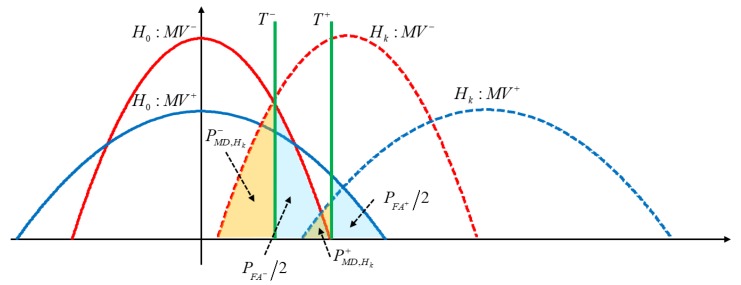
Probability density function of two monitoring values; the region shaded in orange represents the probability of missed detection, and the sky-blue region represents half of the false-alarm probability.

Figure 5 shows the PDF of two independent MVs under the above hypotheses. Considering a given threshold, there is a possibility that the MV exceeds the threshold when a cycle slip does not occur. Such an event is termed as a false alarm (FA). On the other hand, there is the possibility that the MV does not exceed the threshold when cycle slips are actually present. That is, the shifted bias under the cycle-slip hypothesis can be smaller than the given threshold. Such an event is termed as missed detection (MD) [28,29,30]. FA and MD can be derived as follows:

Probability of false alarm for each MV:

(23) $$P_{FA}^{-}=P\left(\left|MV^{-}\right|\geq T^{-}|H_{0}\right)=2\left(1-\Phi \left(\frac{T^{-}}{\sigma_{MV^{-}}}\right)\right),\;$$

(24) $$P_{FA}^{+}=P\left(\left|MV^{+}\right|\geq T^{+}|H_{0}\right)=2\left(1-\Phi \left(\frac{T^{+}}{\sigma_{MV^{+}}}\right)\right).\;$$

Probability of missed detection for each MV:

(25) $$P_{MD,H_{k}}^{-}=P\left(\left|MV^{-}\right|<T^{-}|H_{k}\right)=\Phi \left(\frac{T^{-}-\mu_{k}^{-}}{\sigma_{MV^{-}}}\right),\;$$

(26) $$P_{MD,H_{k}}^{+}=P\left(\left|MV^{+}\right|<T^{+}|H_{k}\right)=\Phi \left(\frac{T^{+}-\mu_{k}^{+}}{\sigma_{MV^{+}}}\right),\;$$

(27) $$where\;\Phi (x)=\int_{-\infty }^{x}\frac{1}{\sqrt{2\pi }}\exp\left(-\frac{1}{2}z^{2}\right)dz.\;$$

Since cycle slips are detected using two complementary and independent MVs, the total probability of FA is determined as the sum of the FA rate of each MV. On the other hand, the total probability of MD can be calculated by the product of the MD rate of each MV.

Total probability of false alarm:

(28) $$P_{FA}=P\left(\left|MV^{-}\right|\geq T^{-}\;or\;\left|MV^{+}\right|\geq T^{+}|H_{0}\right)=P_{FA}^{-}+P_{FA}^{+}.\;$$

Total probability of missed detection (under the $H_{k}$ hypothesis):

(29) $$P_{MD,H_{k}}=P\left(\left|MV^{-}\right|<T^{-}\;and\;\left|MV^{+}\right|<T^{+}|H_{k}\right)=P_{MD,H_{k}}^{-}\times P_{MD,H_{k}}^{+}.\;$$

#### 2.3.2. Detection Threshold Determination

A good detection algorithm has small probabilities of both FA and MD; however, these probabilities are correlated with each other by the detection threshold. If we determine the threshold to minimize FA rate, the probability of MD should be increased and vice versa. Obviously, a threshold of high-integrity and safe detection should be determined to minimize the probability of MD, which is more critical and important than the FA rate [28,29,30]. Typically, a detection threshold of integrity monitors or a fault-detection algorithm is determined on the basis of an FA probability allocated from the continuity requirement of the system. The MD probability is computed by a given threshold. Next, we verify whether the MD rate falls within the required level. If the probability of MD is greater than the desired integrity requirement, we need to re-design the detection threshold based on the re-allocated FA rate [5,45].

According to the above threshold-determination scheme, we allocated the same probability of FA for each MV as 5.0 × 10^−6^. That is, the desired total FA probability is 10^−5^. The corresponding confidence level *K_FA_* is 4.565 that determined by the function “norminv” representing the normal inverse CDF in MATLAB. In conclusion, the proposed cycle-slip detection method introduced in Equation (11) can be re-expressed with the determined threshold values as follows:

(30) $$\begin{array}{ll} & \left|MV^{-}\right|>T^{-}=K_{FA}^{-}\cdot \sigma_{MV^{-}}=0.069\;m/s^{2}\\ or & \left|MV^{+}\right|>T^{+}=K_{FA}^{+}\cdot \sigma_{MV^{+}}=0.078\;m/s^{2}\\ where & K_{FA}^{-}=\mathrm{norminv}\left(1-P_{FA}^{-}/2,0,1\right)=4.565\;(∵P_{FA}^{-}=5.0\;\times \;10^{-6})\\ & K_{FA}^{+}=\mathrm{norminv}\left(1-P_{FA}^{+}/2,0,1\right)=4.565\;(∵P_{FA}^{+}=5.0\;\times \;10^{-6}) \end{array}.$$

#### 2.3.3. Probability of Missed Detection for Insensitive Cycle-Slip Pairs

**Figure 6:**
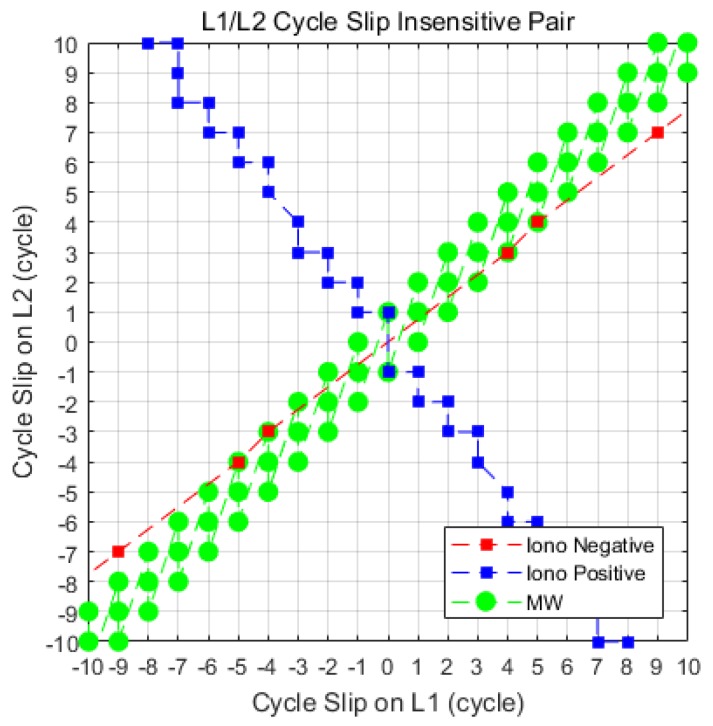
L1/L2 insensitive cycle-slip pairs; the red square points represent the insensitive pairs of the IN combination, the blue square points represent that of the IP combination, and the green circle points represent that of the MW combination.

Figure 6 shows the L1/L2 insensitive cycle-slip pairs for each combination. We assumed that there is only a bias effect $\mu_{k}$ for each cycle-slip pair in the detection test domain. The size of the bias must at least be greater than the threshold in order to be detectable. The red square points represent the insensitive pairs satisfying Equation (31) that do not jump significantly on the IN combination. Similarly, the blue square points show the insensitive pairs of cycle slip that satisfy Equation (32) for the IP combination:

(31) $$\left|\frac{1}{\gamma -1}\left(\lambda_{1}\cdot CS_{1}-\lambda_{2}\cdot CS_{2}\right)\right|<T^{-},\;$$

(32) $$\left|\frac{1}{2}\left(\lambda_{1}\cdot CS_{1}+\frac{1}{\gamma }\lambda_{2}\cdot CS_{2}\right)\right|<T^{+}.\;$$

In order to identify the advantage of the proposed method, we have confirmed the insensitive pairs of *MW* combination satisfying condition as follows:

(33) $$\left|\lambda_{WL}\cdot \left(CS_{1}-CS_{2}\right)\right|<4\cdot \sigma_{MW},$$

where the wide-lane (*WL*) wavelength $\lambda_{WL}=0.86\;m$ and the threshold for detection is set to 4 sigma of the standard deviation $\sigma_{MW}\approx 0.4\;m$ that is considering the pseudorange multipath level of CORS receivers [4,10,13]. These insensitive pairs are represented by the green circle points of Figure 6.

As shown in Figure 6, the general method, which employed the IN and MW combination (the red square points and green circle points) as MVs, cannot detect special cycle-slip pairs (4, 3) and (5, 4). The MW combination have a same signs of inclination as the IN combination for insensitive pairs. The pseudorange multipath effect should be smoother for detecting small cycle slips, but there is a limitation because of remaining bias of multipath. On the other hand, the proposed algorithm (the red square points and blue square points) can detect all insensitive cycle-slip pairs since the two complementary combinations have an opposite signs of inclination. Their inclination is completely orthogonal. In other words, they do not share the insensitive pairs with each other.

**Figure 7:**
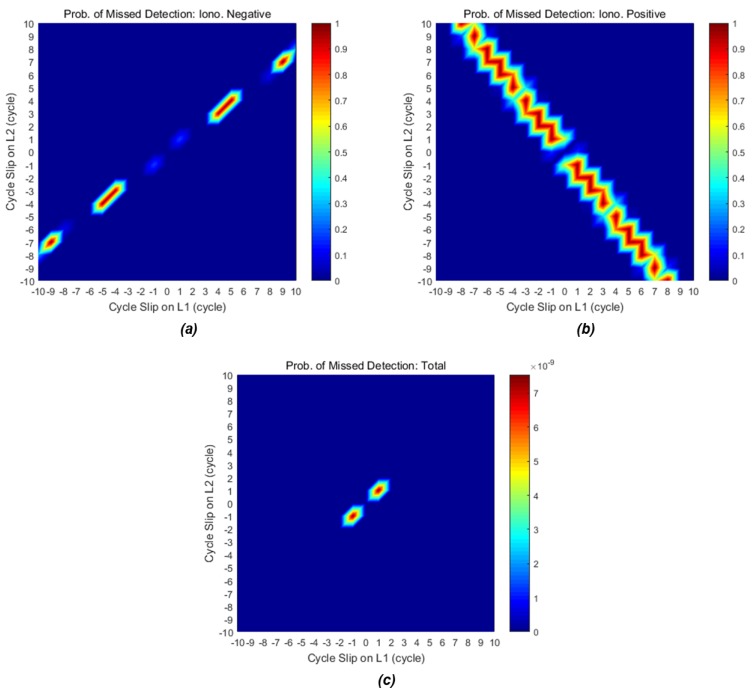
Probability of missed detection for insensitive cycle-slip pairs: (**a**) only the IN combination; (**b**) only the IP combination; (**c**) total probability of missed detection using the two combinations simultaneously. The maximum probability is 7.5 × 10^−9^ when the cycle-slip pair is (1, 1) or (−1, −1).

In order to evaluate the detection performance of the proposed method, we calculate the probability of MD for cycle-slip pairs of each linear combination. The evaluated probability of MD is shown in the color map in Figure 7. Figure 7a represents the probability of MD for IN combination by Equation (25), and Figure 7b represents the probability of MD for IP combination by Equation (26). The cycle slips are easily detectable when the color area is blue, while it is difficult to detect when the color changes toward red. The insensitive cycle-slip pairs for each individual combination have a large MD rate close to one; however, the total probability of MD by the product of the MD rate of each combination is very small because the insensitive pairs can surely be detected by the complementary and orthogonal MVs. For example, probability of MD for pair (1, 1) of IN combination is 0.174. That is, the IN combination may fail to detect the pair (1, 1) with a probability of 17.4%, whereas the pair (1, 1) has a MD probability of 4.3 × 10^−8^ by the IP combination. Therefore, the total probability of MD is 7.5 × 10^−9^ that is the product value of 0.174 and 4.3 × 10^−8^. 

The maximum probability of missed detection is 7.5 × 10^−9^ when the cycle-slip pair is (1, 1) or (−1, −1). The remaining pairs have a much smaller probability. Consequently, our detection method using the acceleration of the IN and IP combinations ensures high-integrity detection with a maximum MD probability of 7.5 × 10^−9^ under the desired total FA probability of 10^−5^. Table 2 summarizes the list of principal insensitive pairs for each combination with their MD probabilities. Even if the cycle-slip pair has an opposite sign, it has the same probability symmetrically.

In addition, we evaluate the probability of MD for general method, which employed the IN and MW combination as MVs, with same strategy. The comparison results of general method and proposed method are summarized in Table 3. As shown in Table 3, the general method may fail to detect some special cycle-slip pairs such as (4, 3), (5, 4), and (9, 7) with a probability more than 40%, while the proposed method can be detect all cycle-slip pairs with improved MD probability. Though it can be applied only stationary observations, we verified that the IP combination can be effectively detect cycle-slip with the IN combination over the MW combination. Therefore, the proposed method is suitable for treating dual-frequency cycle slips on network-based RTK technique.

### 2.4. Cycle-Slip Compensation Method

#### 2.4.1. Cycle-Slip Identification Method

The detected cycle slip should be removed or repaired in order to maintain the integer ambiguity on the carrier phase. In this section, we discuss the method for cycle-slip compensation (or recovery). The first step is cycle-slip identification for estimating the exact size of the cycle slip. We can construct the observation matrix A for estimating the original L1 and L2 cycle slips with the two independent combinations, IN and IP acceleration:

(34) $$\begin{array}{l} z=\left[\begin{array}{c} MV^{-}\\ MV^{+} \end{array}\right]=\left[\begin{array}{cc} \frac{1}{\gamma -1}\lambda_{1} & \frac{-1}{\gamma -1}\lambda_{2}\\ \frac{1}{2}\lambda_{1} & \frac{1}{2\gamma }\lambda_{2} \end{array}\right]\left[\begin{array}{c} CS_{1}\\ CS_{2} \end{array}\right]+\left[\begin{array}{c} \varepsilon_{\delta_{T}^{2}\Delta I^{-}}\\ \varepsilon_{\delta_{T}^{2}\Delta I^{+}} \end{array}\right]=Ax+\varepsilon \\ where\;\varepsilon \sim N\left(0,Q_{zz}\right)\;Q_{zz}=diag\left(\sigma_{MV^{-}}^{2},\sigma_{MV^{+}}^{2}\right) \end{array}.$$

Therefore, the float solution of L1 and L2 cycle slips can be obtained by the weighted least-squares estimation as follows:

(35) $$\hat{x}=\left[\begin{array}{c} \begin{array}{c} \hat{CS_{1}} \end{array}\\ \hat{CS_{2}} \end{array}\right]={\left(A^{T}Q_{zz}^{-1}A\right)}^{-1}A^{T}Q_{zz}^{-1}z,\;$$

(36) $$Q_{\hat{x}\hat{x}}={\left(A^{T}Q_{zz}^{-1}A\right)}^{-1}.\;$$

These float solutions are needed to fix an integer solution of cycle slip, and we employ the LAMBDA integer mapping method, which is widely used for ambiguity resolution [31]. The LAMBDA method gives the best integer solution through the integer least squares; that is, the success probability of the LAMBDA method is better than that of the popular integer rounding method [6,46]. Therefore, the LAMBDA method is reasonable for identifying the integer size of cycle slip.

The lower bound (or worst) probability of correct L1/L2 cycle-slip identification can be calculated from the success rate of bootstrapping (or conditional integer rounding) as follows [6,46]:

(37) $$P_{s}=P\left(\overset{⌣}{x}=x\right)=P\left(\left|\hat{x}-x\right|\leq \frac{1}{2}\right)=\prod_{i=1}^{2}\left\{2\cdot \Phi \left(\frac{1}{2\sigma_{CS_{i}}}\right)-1\right\},\;$$

where the symbol Φ is the CDF of the standard normal distribution given by Equation (27), the conditional standard deviations $\sigma_{CS_{i}}$ (i=1,  2 for each frequency) are the square roots of the entries of the diagonal matrix obtained from the LDL decomposition of the covariance matrix $Q_{\hat{x}\hat{x}}$ of least-squares estimation.

In order to evaluate the performance of the proposed identification method, we calculate the probability of failure, which refers to the probability that the float solution is pulled to a wrong integer cycle slip. Finally, our identification method ensures high-integrity performance with the upper-bound failure rate $P_{f}=1-P_{s}=1.4\;\times \;10^{-8}$.

#### 2.4.2. Cycle-Slip Validation Method

After the cycle-slip identification, we conduct a validation test to ensure whether the actual cycle slip occurred. The outlier, which corresponds to impulse anomaly with an error of an arbitrary size at a specific epoch, appear similar jump as the cycle slip in the time-difference domain. Therefore, we should distinguish them through the validation test.

Once the size of the cycle slip is determined by the identification method, the two MVs used in the cycle-slip detection can be repaired. These repaired MVs cannot exceed the detection threshold if the actual cycle slip is compensated correctly. Therefore, the cycle slips can be validated when the following condition is satisfied:

(38) $$\begin{array}{ll} & \left|MV_{repaired}^{-}\right|<T^{-}=K_{FA}^{-}\cdot \sigma_{MV^{-}}=0.069\;m/s^{2}\\ and & \left|MV_{repaired}^{+}\right|<T^{+}=K_{FA}^{+}\cdot \sigma_{MV^{+}}=0.078\;m/s^{2} \end{array}.$$

The identified cycle slip passing the validation test is compensated and employed as the normal carrier-phase observation, while a phase anomaly that does not satisfy the above condition is considered an outlier that must be removed.

**Figure 8:**
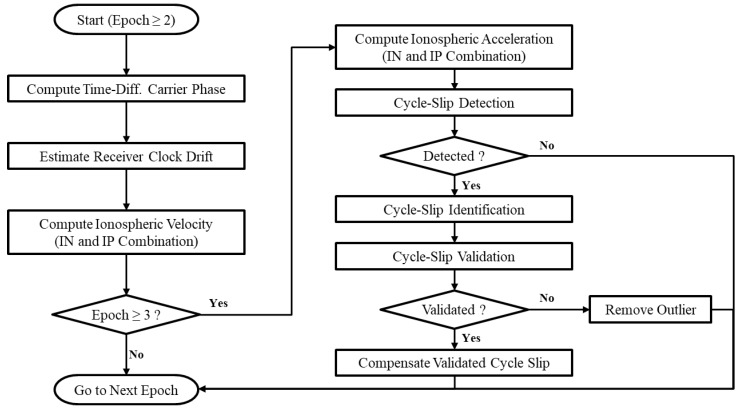
Block diagram of the overall cycle-slip detection and compensation algorithm.

A block diagram of the proposed cycle-slip detection and compensation algorithm is shown in Figure 8.

## 3. Algorithm Test Results

### 3.1. Algorithm Test Environment

**Figure 9:**
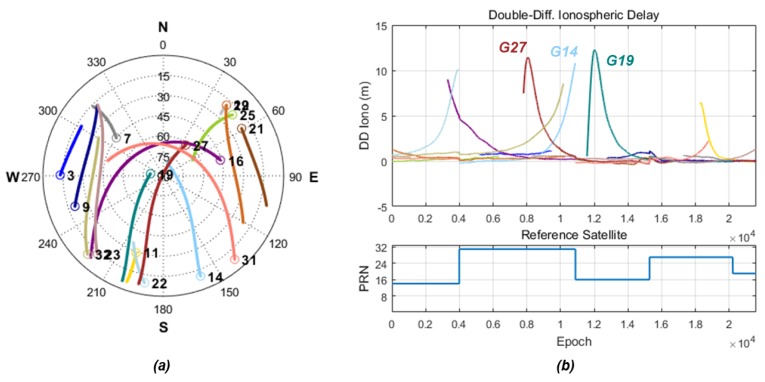
(**a**) Sky plot in the CHJU station; (**b**) double-differenced ionospheric delay (RTK results).

The proposed algorithm is verified using GPS carrier-phase measurements collected under a severe geomagnetic storm that occurred on 17 March 2015. The test environment is summarized in Table 4. We collected the GPS dual-frequency carrier phase with 1-s intervals from the GANH (37.72° N, 126.39° E) and CHJU (33.51° N, 126.53° E) stations of NGII in Korea. Their locations are shown in Figure 2, and the baseline length between them is 467 km. The maximum ionospheric disturbance was observed between 15:00:00 and 21:00:00 local time in the Korean region (UTC + 9). Figure 9 represents the satellite geometry during the test time in the CHJU station and the double-differenced ionospheric delay obtained from the long-baseline RTK system. As can be seen in this figure, double-differenced ionospheric delay is dramatically disturbed at a low elevation.

Therefore, we conducted the algorithm test using the low-elevation data of G14, G19, and G27, which are specifically affected by the ionospheric disturbance. Practically, cycle slips frequently occur in the low-elevation data owing to the low signal-to-noise ratio and the possibility of signal blockage [4,7]. First, we confirmed that no real cycle slip occurred in these target data by pre-processing using Bernese GNSS software. Next, we forcibly inserted the simulated cycle slips, which are the principal insensitive pairs listed on Table 2, every 100 epochs into the raw observations.

### 3.2. Data Test Results

**Figure 10:**
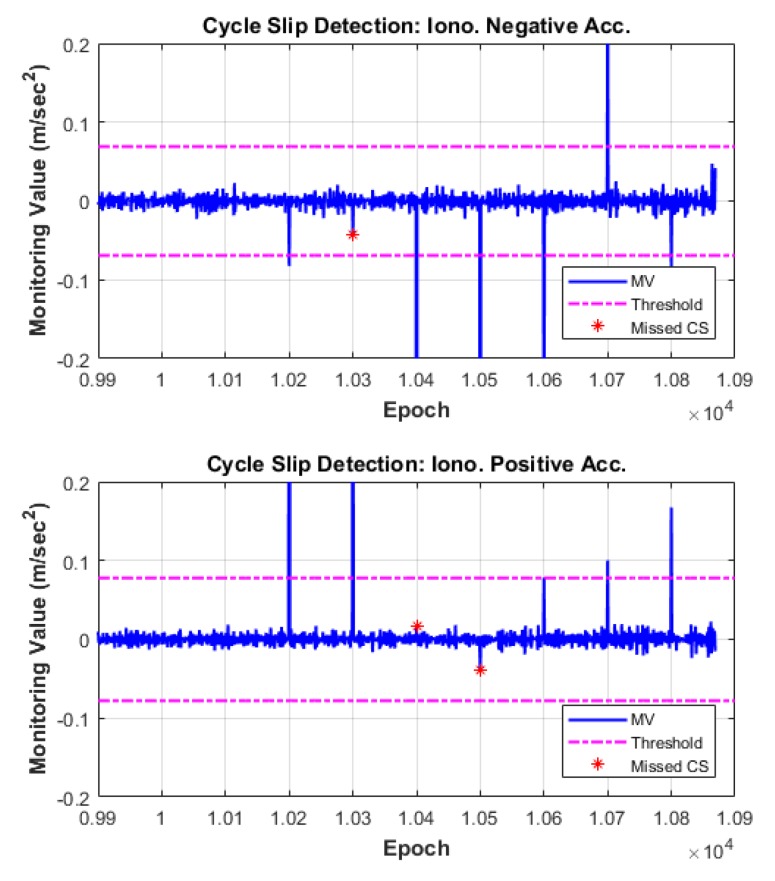
Cycle-slip detection results for G14.

**Figure 11:**
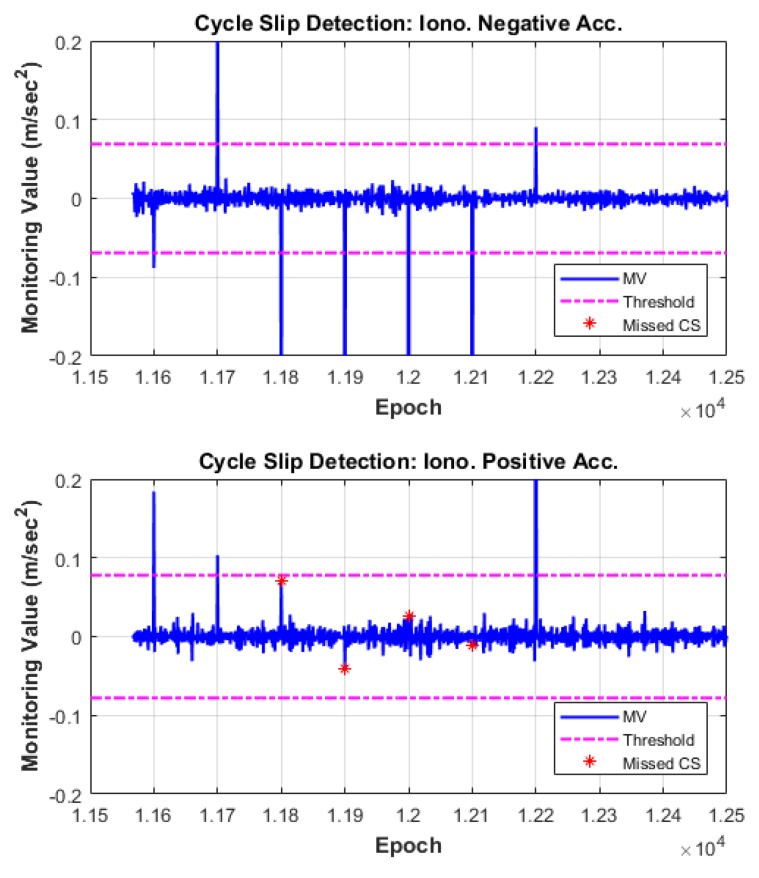
Cycle-slip detection results for G19.

**Figure 12:**
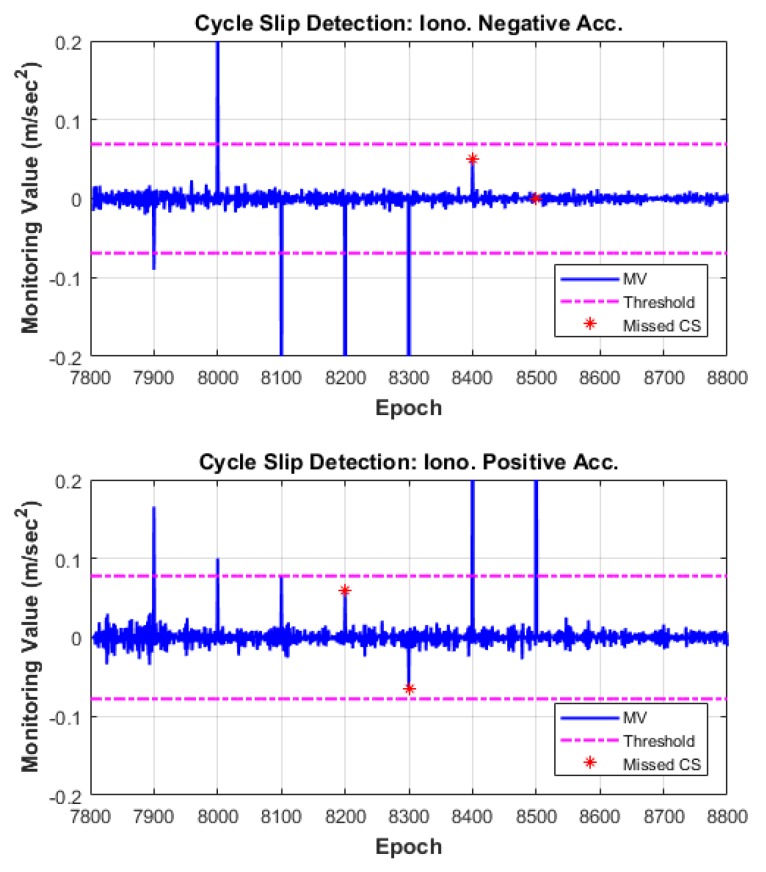
Cycle-slip detection results for G27.

We verified the performance of the proposed cycle-slip detection and compensation algorithm through the observation data in which simulated cycle slips were added. The algorithm test results are summarized in Table 5, and the time series of MVs of each satellite are illustrated in Figure 10, Figure 11 and Figure 12. In these figures, the top subplot represents IN acceleration, and the bottom subplot represents IP acceleration. The blue line shows the MV and the dash-single-dotted magenta line indicates the threshold for cycle-slip detection. The red star points show the missed cycle slip, which correspond to the insensitive pairs for each combination.

As can be seen from the results, all of the cycle slips including the pair (1, 1) for which the probability of MD has the maximum value are correctly detected by the two independent and complementary MVs, IN and IP acceleration. For example, the cycle-slip pair (5, 4), one of the most challenging pairs to detect in many previous studies, is not detected by the IN combination but is successfully detected using the IP combination. In contrast, the insensitive pairs in the IP combination are apparently detected under the IN combination.

The proposed algorithm also correctly identifies the original size of L1 and L2 cycle slips. Columns 6 and 7 of Table 5 list the float solutions of the estimated cycle slips. These float solutions are mapped to integer values by the LAMBDA technique and reliably compensated from the validation test.

## 4. Conclusions

This paper proposed a cycle-slip detection and compensation algorithm using only dual-frequency carrier-phase stationary observations. In order to detect and repair cycle slips with high-integrity performance, two complementary ionospheric combinations called the IN and IP combinations are employed. They can successfully detect all of the cycle slips since two L1/L2 combinations combine cycle slips with opposite signs for uniquely detecting insensitive pairs. Furthermore, we treat the second-order time-differenced observation as the MVs to reduce the impact of ionospheric delay even for long-baseline distance of CORS network. We verified that the actual error distributions of the MVs under high ionospheric storm are sufficiently bounded and properly assumed at the 10^−7^-level probability of the normal Gaussian distribution from a theoretical analysis. The detection threshold is determined by the total desired probability of FA of 10^−5^. Consequently, our detection method ensures high-integrity performance with a maximum probability of MD of 7.5 × 10^−9^. We also introduced a LAMBDA-based cycle-slip identification method, for which the upper-bound failure rate is 1.4 × 10^−8^. Finally, through a cycle-slip validation test, we can correctly compensate for a real cycle slip and distinguish it from outliers. An algorithm verification test was conducted using actual data collected under a severe ionospheric storm. As a result, all artificially inserted cycle slips, including small cycle slips, were successfully detected and compensated for. In summary, we demonstrated that the proposed method is suitable for monitoring dual-frequency cycle slips on network RTK systems, which should generate high-precision corrections with high-integrity information.

In this paper, although we described an algorithm based on the SD between stationary receivers for applying the network-based RTK technique, we expect that the method can be modified and extended for many dynamic applications through INS integration or Doppler aiding. 

## Figures and Tables

**Table 1:** Statistics of actual error distribution of the monitoring values.

Combination	Number of Sample	Actual Sample Sigma	Theoretical Sigma	Bounding Scale Factor
Negative (IN)	991,7379	4.6 mm/s^2^	15.1 mm/s^2^	3.3
Positive (IP)	991,7379	4.9 mm/s^2^	17.1 mm/s^2^	3.5

**Table 2:** Principal insensitive pairs of cycle slip with the probability of missed detection of proposed method (any probability less than 10^−100^ is entered as zero in the table).

Cycle Slip (Cycle)	Iono. Negative		Iono. Positive		Total
	Bias (m)	P_MD_	Bias (m)	P_MD_	P_MD_
(1, 0)	0.294	3.1 × 10^−50^	0.095	0.156	4.9 × 10^−51^
(0, 1)	0.378	1.9 × 10^−92^	0.074	0.588	1.1 × 10^−92^
(1, 1)	0.083	0.174	0.169	4.3 × 10^−8^	7.5 × 10^−9^
(−1, 1)	0.672	0	0.021	1.000	0
(−1, 2)	1.049	0	0.053	0.928	0
(−2, 2)	1.343	0	0.042	0.982	0
(−2, 3)	1.721	0	0.032	0.996	0
(−3, 3)	2.015	0	0.063	0.809	0
(−3, 4)	2.392	0	0.011	1.000	0
(−4, 5)	3.064	0	0.001	1.000	0
(4, 3)	0.044	0.951	0.603	0	0
(5, 4)	0.039	0.976	0.772	0	0
(8, 6)	0.088	0.104	1.206	0	0
(9, 7)	0.005	1.000	1.375	0	0
(10, 8)	0.078	0.270	1.545	0	0

**Table 3:** Comparison results of the probability of missed detection (any probability less than 10^−100^ is entered as zero in the table).

Cycle Slip (Cycle)	General Method (IN + MW)	Proposed Method (IN + IP)
(1, 0)	6.2 × 10^−61^	4.9 × 10^−51^
(0, 1)	0	1.1 × 10^−92^
(1, 1)	6.2 × 10^−3^	7.5 × 10^−9^
(4, 3)	0.497	0
(5, 4)	0.613	0
(8, 6)	9.5 × 10^−4^	0
(9, 7)	0.401	0
(10, 8)	5.9 × 10^−3^	0

**Table 4:** Algorithm test environment.

Date	17 March 2015
Time	UTC 06:00:00~11:59:59 (6 h)
Interval	1 s
Baseline	GANH–CHJU (467 km)
Receiver	Trimble NetR9
Antenna	Trimble Zephyr (TRM59800)
Kp index	8—(Daily maximum)

**Table 5:** Cycle-slip detection and compensation results.

PRN	Epoch	El (°)	Inserted Cycle Slip (Cycle)	Monitoring Value (Meter)		Estimated CS (Cycle)		Validated Cycle Slip (cycle)
				MV^−^	MV^+^	L1 CS	L2 CS	
G14	10200	14.81	(10, 8)	−0.083	1.553	10.029	8.029	(10, 8)
	10300	14.09	(5, 4)	−0.043	0.768	4.996	4.006	(5, 4)
	10400	13.37	(−3, 4)	−2.403	0.024	−2.943	4.062	(−3, 4)
	10500	12.65	(−2, 2)	−1.341	−0.039	−1.998	2.011	(−2, 2)
	10600	11.94	(0, 1)	−0.386	0.083	0.030	1.037	(0, 1)
	10700	11.22	(1, 0)	0.289	0.101	1.017	0.020	(1, 0)
	10800	10.52	(1, 1)	−0.084	0.171	1.013	1.015	(1, 1)
G19	11600	5.99	(1, 1)	−0.088	0.183	1.057	1.072	(1, 1)
	11700	6.67	(1, 0)	0.291	0.103	1.017	0.012	(1, 0)
	11800	7.36	(0, 1)	−0.385	0.070	−0.051	0.979	(0, 1)
	11900	8.05	(−1, 1)	−0.669	−0.041	−1.089	0.935	(−1, 1)
	12000	8.74	(−2, 3)	−1.722	0.027	−2.046	2.942	(−2, 3)
	12100	9.44	(−4, 5)	−3.074	−0.011	−4.020	5.015	(−4, 5)
	12200	10.14	(8, 6)	0.091	1.193	7.956	5.952	(8, 6)
G27	7900	7.83	(1, 1)	−0.090	0.166	0.941	0.948	(1, 1)
	8000	8.52	(1, 0)	0.296	0.100	1.003	−0.014	(1, 0)
	8100	9.20	(0, 1)	−0.378	0.078	0.007	1.003	(0, 1)
	8200	9.90	(−1, 2)	−1.047	0.059	−0.980	2.010	(−1, 2)
	8300	10.59	(−3, 3)	−2.020	−0.063	−3.011	2.981	(−3, 3)
	8400	11.29	(4, 3)	0.051	0.610	4.019	3.002	(4, 3)
	8500	11.99	(9, 7)	0.000	1.383	9.015	7.014	(9, 7)
